# Supplementary material for: Selective Detection of Penicillin G Antibiotic in Milk by Molecularly Imprinted Polymer-Based Plasmonic SPR Sensor
Source: Biomimetics (Basel). 2021 Dec 14;6(4):72. doi: 10.3390/biomimetics6040072 (PMC8698653; doi:10.3390/biomimetics6040072)
Supplement: Supplementary file 1 [file biomimetics-06-00072-s001.zip › biomimetics-1484235-supplementary.pdf]

**Supplementary Material**

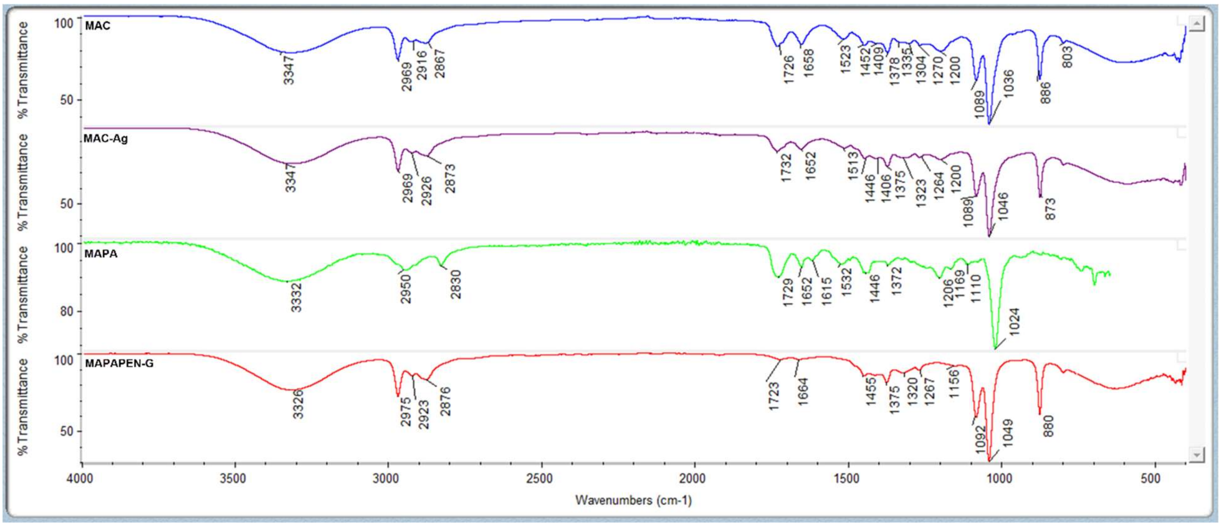


**Supplementary Figure S1.** FTIR-ATR spectrum of MAC monomer and MAC-Ag pre-complex; FTIR-ATR spectrum of MAPA monomer and MAPAPEN-G pre-complex.
